# Diagnostic performance of contrast-enhanced ultrasound in distinguishing lymphoma from non-lymphoma lesions in salivary gland: a retrospective study

**DOI:** 10.3389/fonc.2026.1840807

**Published:** 2026-05-29

**Authors:** Zenghui Liang, Yanan Zhao, Tian Han, Chen Qiu, Minqiang Pan, Qing Wen, Pintong Huang

**Affiliations:** Department of Ultrasound, The Second Affiliated Hospital of Zhejiang University School of Medicine, Hangzhou, Zhejiang, China

**Keywords:** contrast-enhanced ultrasound, differential diagnosis, non-lymphomatous, salivary gland lymphoma, ultrasound

## Abstract

**Background:**

Salivary gland lymphoma is a rare malignancy that is difficult to differentiate from other salivary gland lesions preoperatively. This study aimed to evaluate the diagnostic value of conventional ultrasound (US) and contrast-enhanced ultrasound (CEUS) in distinguishing salivary gland lymphoma from non-lymphoma lesions.

**Methods:**

This retrospective study included patients with salivary gland lesions who underwent ultrasound-guided biopsy or surgical resection at our institution between January 2019 and December 2024. US and CEUS features were compared between lymphoma and non-lymphoma lesions. Multivariate logistic regression and receiver operating characteristic (ROC) curve analyses were performed to identify independent predictors and evaluate diagnostic performance.

**Results:**

A total of 329 patients were included, comprising 36 salivary gland lymphomas and 293 non-lymphoma lesions. Significant differences were observed in lesion number, posterior echo, cystic components, color doppler flow imaging (CDFI), enhancement degree, enhancement pattern, early wash-in, and early wash-out (all *p* < 0.05). Multivariate analysis identified multiple lesions, posterior echo enhancement, non-centripetal enhancement, early wash-in, and early wash-out as independent predictors of salivary gland lymphoma. The combined US and CEUS model achieved a sensitivity of 58.3%, specificity of 98.0%, and accuracy of 93.6%, with an area under the ROC curve (AUC) of 0.950.

**Conclusion:**

These findings demonstrate that CEUS can serve an adjunct tool for the differential diagnosis of salivary gland lymphoma and non-lymphoma.

## 1 Introduction

Salivary gland lymphoma is a rare malignant lesion, accounting for approximately 2% of all salivary gland neoplasms (1, 2). The two most prevalent histological subtypes of salivary lymphoma are mucosa-associated lymphoid tissue (MALT) lymphoma and follicular lymphoma (FL) (3, 4), both of which exhibit distinct pathological features and have a more favorable prognosis than other malignancies. Salivary gland lymphomas are usually asymptomatic. Clinical presentation typically occurs with the onset of facial symptoms, including pain, facial paralysis, or rapid tumor growth, prompting medical consultation (5). However, as patients with these symptoms often first visit the Department of Stomatology instead of the Departments of Hematology, many cases are initially treated as other salivary gland lesions (6).

Few studies have investigated preoperative imaging modalities for salivary gland lymphoma. Nearly 60% of patients were diagnosed using surgical procedures (7). Ultrasound is the primary imaging modality for evaluating salivary gland lesions. Conventional ultrasound (US) and color doppler flow imaging (CDFI) provide detailed morphological and vascular characteristics of the glands, respectively. US is noninvasive, readily available, and easy to perform, and has been unanimously recognized by experts in diagnosing, evaluating, and monitoring salivary gland lesions, such as Sjögren’s syndrome (SS) (8). Furthermore, ultrasound-guided core needle biopsy (CNB) can ensure the acquisition of adequate histological samples from patients with SS suspected of having lymphoma.

However, the capacity of US and CDFI to differentiate salivary gland masses remains limited (9, 10). Contrast-enhanced ultrasound (CEUS), employing microbubble-based contrast agents, facilitates a more accurate and noninvasive assessment of the microvascular architecture, thereby playing a crucial role in distinguishing between benign and malignant salivary gland lesions (11–15). In recent years, CEUS has emerged as a valuable imaging modality for the differential diagnosis of focal salivary gland lesions. However, there are relatively few studies on its diagnostic effect on salivary gland lymphomas. Therefore, this study aimed to evaluate the differences in US and CEUS characteristics between salivary gland lymphoma and non-lymphoma lesions. The study aimed to summarize the differences between the US and CEUS features of 36 cases of salivary gland lymphoma and 293 cases of non-lymphoma lesions to avoid unnecessary surgical complications.

## 2 Materials and methods

### Patients

**Figure 1:**
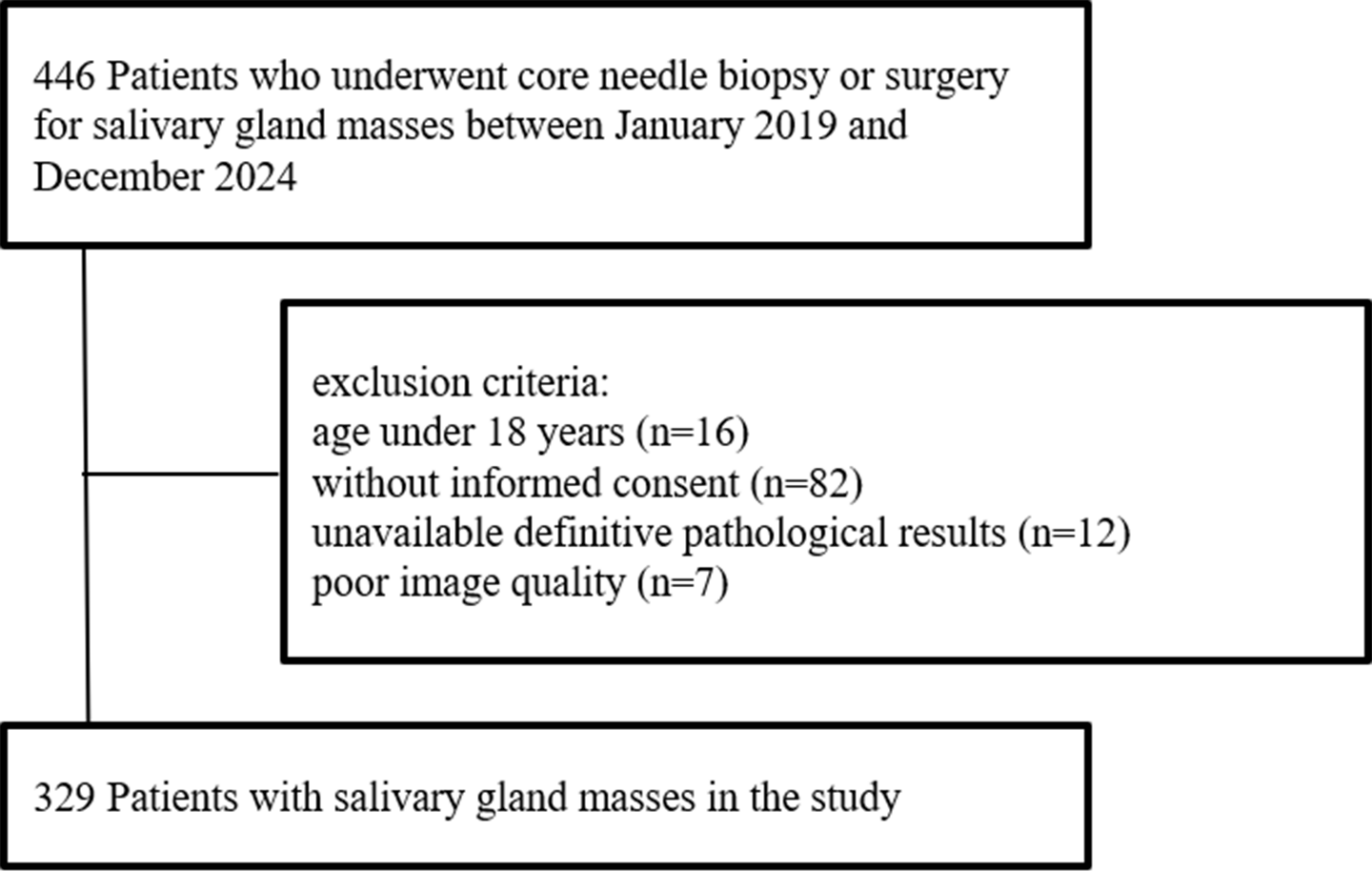
Inclusion and exclusion flowchart.

This retrospective study was approved by the Human Research Ethics Committee of the Second Affiliated Hospital of Zhejiang University School of Medicine (No.2026-0065). All patients provided informed consent before CEUS. From January 2019 to December 2024, 446 patients who underwent core needle biopsy or surgery for salivary gland masses were enrolled in this study. The inclusion criteria were: (1) age ≥18 years; (2) complete clinical and imaging records; (3) availability of conventional US and CEUS examinations; and (4) histopathological confirmation obtained by core needle biopsy or surgical resection. The exclusion criteria were: (1) age < 18 years; (2) lack of informed consent; (3) unavailable definitive pathological results; and (4) poor image quality. Sixteen patients aged < 18 years, 82 patients without informed consent, 12 patients without a definitive pathological diagnosis, and 7 patients with poor image quality were excluded. A total of 329 cases were analyzed, including 36 cases of salivary gland lymphoma and 293 cases of non-lymphoma lesions. **Figure 1** presents a flowchart of the study’s inclusion and exclusion criteria.

### Imaging protocol

US and CEUS examinations were performed using Mindray Resona 7, Mindray Resona 9, and Mindray A20 ultrasound systems (Mindray, Shenzhen, China) equipped with L11–3 and L14–5 transducers. During both US and CEUS examinations, each patient was placed in the supine position with the neck hyperextended. US examination was first conducted to scan the salivary glands and identify the number and location of the masses. The following sonographic features of each mass were analyzed: maximum diameter, shape, orientation, internal and posterior echo, cystic components, and vascularity on color doppler imaging. Subsequently, CEUS was performed by administering a 2.4 mL bolus of microbubble contrast agent (SonoVue, Bracco, Italy) via the antecubital vein, followed by a 5 mL flush with 0.9% saline. Real-time CEUS imaging was acquired continuously for 180 seconds post-injection. The imaging parameters were set as follows: mechanical index, 0.06–0.08; gain, 50–65 dB; and frame rate, 10–15 frames per second. The entire CEUS cine loop was stored on the scanner’s hard drive for subsequent qualitative assessment of the enhancement pattern of each lesion.

### Analysis of the US image

The ultrasonographic features of each salivary gland lesion were analyzed, including: (1) Number of lesions: solitary or multiple; (2) Location: parotid or submandibular gland; (3) Shape: regular or irregular; (4) Growth orientation: parallel (wider-than-tall) or expansive (taller-than-wide); (5) Internal echo: even or uneven; (6) Posterior echo: enhancement or no enhancement; (7) Cystic component: yes or no; and (8) CDFI pattern, classified according to the Adler semiquantitative scale: grade 0, no blood flow within the mass; grade I, minimal flow with 1–2 punctate or rod-like vessels; grade II, moderate flow with 3–4 punctate vessels or a single long vessel (length close to or exceeding the lesion radius) traversing the mass; grade III, abundant flow with≥5 punctate vessels or 2 long vessels.

### Analysis of the CEUS image

The stored dynamic CEUS cine loops were retrospectively analyzed to assess the following parameters: (1) Enhancement degree: signal intensity after contrast injection, categorized as marked hyperenhancement or no hyperenhancement (compared with the adjacent normal salivary gland tissue at the same depth); (2) Enhancement pattern: defined as centripetal enhancement (if enhancement progressed from the periphery toward the center) or non-centripetal enhancement (simultaneous enhancement of both the peripheral and central portions of the lesion or enhancement progressing from the center toward the periphery, rather than the typical peripheral-to-central enhancement pattern); (3) Enhancement uniformity: uniform or non-uniform; (4) Enhancement margin: clear (>50% of the enhanced area had a sharp border) or blurred (<50% of the enhanced area had a sharp border or the border was unclear); (5) Size of enhanced lesion: assessed as expansion or non-expansion compared with the pre-contrast measurement; (6) Wash-in pattern: whether the lesion began to enhance earlier, synchronously, or later than the surrounding normal gland; (7) Wash-out pattern: whether the lesion began to wash out earlier, synchronously, or later than the surrounding normal gland.

The US and CEUS images were retrospectively analyzed by two ultrasonologists, each with over 10 years of experience in ultrasonography. The images were jointly reviewed, and any discrepancies were resolved by consensus. Both reviewers were blinded to the final pathological diagnosis.

### Statistical analysis

All statistical analyses were performed using SPSS version 25.0 (IBM Corp., Chicago, IL, USA). Continuous variables were expressed as mean ± standard deviation and compared using the independent samples *t*-test. Categorical variables were presented as percentages and analyzed with the Chi-square test or the continuity-corrected Chi-square test when the sample size was >40 and at least one expected frequency was between 1 and 5. The Mann-Whitney U test was used for ordinal or non-normally distributed variables. Multivariate binary logistic regression was employed to analyze US and CEUS indicators and establish a predictive model. A two-sided *p* < 0.05 was considered statistically significant. Receiver operating characteristic (ROC) curve analysis of the parameters of combined US and CEUS was performed to differentiate diagnosis of salivary gland lymphoma and non-lymphoma.

## 3 Results

### Clinical and pathological characteristics

**Figure 2:**
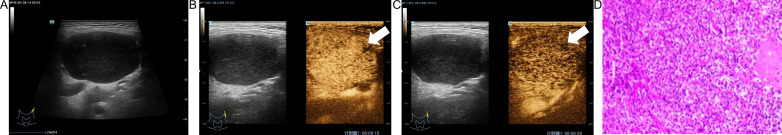
Mucosa-associated lymphoid tissue (MALT) lymphoma of the left parotid gland. A 70-year-old male patient presented with a progressive enlargement of a mass near his left ear for more than 6 months. **(A)** The ultrasound examination revealed a well-defined mass with a regular shape and enhanced posterior echo. **(B)** Early phase of CEUS showed non-centrifugal, rapid wash-in, and diffuse hyperenhancement (white arrow). **(C)** Middle phase of CEUS showed rapid wash out (white arrow). **(D)** H&E staining (original magnification X40; section thickness, 5 μm).

**Figure 3:**
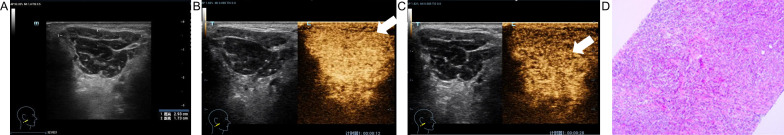
Diffuse large B-cell lymphoma of the right parotid gland. A 58-year-old male patient presented with a painless mass in the right parotid gland for over a month, which gradually increased in size. **(A)** The ultrasound examination revealed an ill-defined mass with an irregular shape and enhanced posterior echo. **(B)** Early phase of CEUS showed non-centrifugal, rapid wash-in, and diffuse hyperenhancement with blurred enhancement margin (white arrow). **(C)** Middle phase of CEUS showed rapid wash out (white arrow). **(D)** H&E staining (original magnification X20; section thickness, 5 μm).

**Figure 4:**
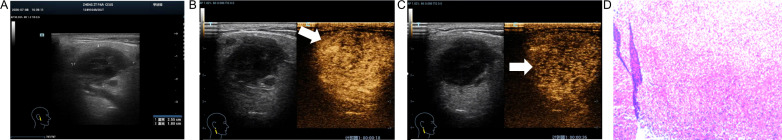
Follicular lymphoma (FL) of the right parotid gland. A 68-year-old male patient complained of discomfort in the right ear for over 4 months. **(A)** The ultrasound examination revealed a well-defined, irregularly shaped mass with an enhanced posterior echo. **(B)** The early phase of CEUS showed non- centrifugal, rapid wash-in, and diffuse hyperenhancement (white arrow). **(C)** The middle phase of CEUS showed rapid washout in 23 seconds (white arrow). **(D)** H&E staining (original magnification X20; section thickness, 5 μm).

**Figure 5:**
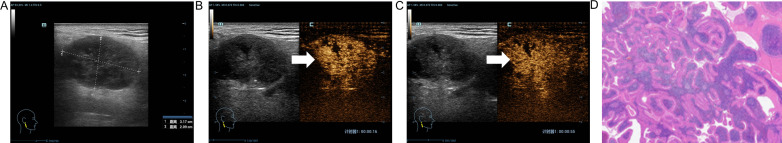
Warthin tumor (WT) of the right parotid gland. A 60-year-old male patient discovered a mass in the anterior part of his right ear after about a month. **(A)** The ultrasound examination revealed a well-defined, regularly shaped mass with an enhanced posterior echo. **(B)** The early phase of CEUS showed centrifugal, slowly wash-in, and hyperenhancement (white arrow). **(C)** The late phase of CEUS showed a slow washout (white arrow). **(D)** H&E staining (original magnification X20; section thickness, 5 μm).

**Figure 6:**
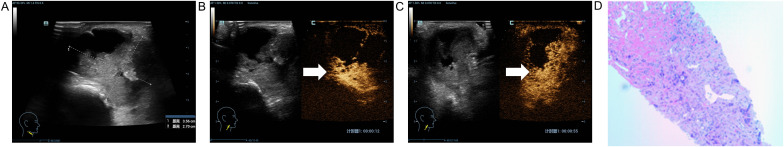
Salivary ductal carcinoma of the right parotid gland. A 60-year-old male patient found a mass in the right parotid gland for over 4 months. **(A)** The ultrasound examination revealed an ill-defined, irregularly shaped mass with an enhanced posterior echo. **(B)** The early phase of CEUS showed non- centrifugal, rapid wash-in, and hyperenhancement (white arrow). **(C)** The late phase of CEUS showed a slow washout (white arrow). **(D)** H&E staining (original magnification X20; section thickness, 5 μm).

The 36 patients with salivary gland lymphoma ranged in age from 26 to 93 years, with a mean age of 60.52 ± 16.41 years. The 293 patients with non-lymphoma lesions ranged in age from 19 to 91 years, with a mean age of 56.84 ± 16.74 years. The proportion of patients with multiple lesions was significantly higher in the lymphoma group (47.2%, 17/36) than in the non-lymphoma group (12.7%, 37/293) (*p* < 0.001). No statistically significant differences were observed between the two groups in terms of sex, age, and lesion location (*p* = 0.131, 0.213, 0.723, respectively). Detailed information is presented in **Table 1**. A total of 329 patients with salivary gland lesions were included in this study. The pathological diagnosis for all cases was confirmed by ultrasound-guided CNB or surgical resection. The lesions were located in the parotid gland (n=267) or the submandibular gland (n=62). The cohort comprised 36 (10.9%) lymphomas and 293 (89.1%) non-lymphomatous lesions. The lymphoma group consisted of 17 cases of mucosa-associated lymphoid tissue (MALT) (**Figure 2**), 15 cases of diffuse large B-cell lymphoma (DLBCL) (**Figure 3**), and 5 cases of follicular lymphoma (FL) (**Figure 4**). The non-lymphoma group included both benign and malignant lesions. Benign lesions (n=205) (**Figure 5**) included adenolymphoma (n=55), pleomorphic adenoma (n=76), chronic inflammatory lesions (n=60), basal cell adenoma (n=7), lymphoepithelial cyst (n=4), eosinophilic cell tumor (n=2), Kimura disease (n=1), and nerve sheath tumor (n=1). Malignant lesions (n=88) (**Figure 6**) included squamous cell carcinoma (n=20), salivary ductal carcinoma (n=15), adenoid cystic carcinoma (n=13), metastatic carcinoma (n=10), mucoepidermoid carcinoma (n=8), adenocarcinoma (n=6), acinar cell carcinoma (n=5), lymphoepithelial carcinoma (n=5), poorly differentiated carcinoma (n=3), and myoepithelial carcinoma (n=2). The detailed pathological characteristics of both groups are summarized in **Table 2**.

**Table 1:** Detailed clinical-pathologic characteristics of lesions in the salivary gland(n=329).

Characteristic	Overall (n = 329)	Lymphoma (n = 36)	No-lymphoma (n = 293)	χ2/t value	*p*
Gender					
Male	185(56.2%)	16(44.4%)	169(57.6%)	2.282*	0.131
Female	144(43.8%)	20(55.6%)	124(42.3%)		
Age (y)		60.53 ± 16.41	56.84 ± 16.74	-1.249‡	0.213
Number					
Single	275(83.5%)	19(52.7%)	256(87.3%)	27.967*	<0.001
Multiple	54(16.5%)	17(47.3%)	37(12.7%)		
Location					
Parotid gland	267(81.1%)	30(83.3%)	237(80.8%)	0.125*	0.723
Submandibular gland	62(18.9%)	6(16.7%)	56(19.2%)		

*, χ2 test; ^‡^, independent *T*-test.

**Table 2:** Histopathological result of lesions in the salivary gland(n=329).

Histopathological result	NO.
Lymphoma(n=36)	
Mucosa-associated lymphoid tissue (MALT) lymphoma	17(47.2%)
Diffuse large B-cell lymphoma (DLBCL)	14(38.9%)
Follicular lymphoma (FL)	5(13.9%)
Non-lymphoma(n=293)	
Benign	
Adenolymphoma	55(18.8%)
Pleomorphic adenoma	76(25.9%)
Chronic inflammatory lesions	60(20.5%)
Basal cell adenomas (BCA)	7(2.4%)
Lymphatic epithelial cyst	4(1.4%)
Eosinophilic cell tumor	2(0.7%)
Kimura disease	1(0.3%)
Nerve sheath tumor	1(0.3%)
Malignant	
Squamous cell carcinoma	20(6.8%)
Salivary ductal carcinoma	15(5.1%)
Adenoid cystic carcinoma	13(4.4%)
Metastatic carcinoma	10(3.4%)
Mucoepidermoid carcinoma	8(2.7%)
Adenocarcinoma	6(2.0%)
Acinar cell carcinoma	5(1.7%)
Lymphoepithelial carcinoma	5(1.8%)
Poorly differentiated carcinoma	3(1.0%)
Myoepithelial carcinoma	2(0.7%)

### Ultrasound characteristics

**Table 3** summarizes and compares US characteristics of 36 salivary gland lymphomas and 293 non-lymphomatous lesions. Statistically significant differences were observed in several US features. The incidence of posterior echo enhancement was significantly higher in lymphomas (88.9%, 32/36) than in non-lymphomas (70.9%, 208/293; *p* = 0.023). Conversely, the presence of a cystic component was significantly less frequent in lymphomas (22.2%, 8/36) than in non-lymphomas (46.1%, 135/293; *p* = 0.006). Vascularity, assessed by color doppler, also differed markedly. The combined proportion of Adler grades 2 and 3 was significantly higher in the lymphoma group (77.8%) than in the non-lymphoma group (48.1%; *p* < 0.001). No statistically significant differences were found between the two groups regarding other US features, including lesion diameter, shape, orientation, internal echo, and calcifications (all *p* > 0.05).

**Table 3:** The US characteristics of lesions in the salivary gland(n=329).

Characteristic	Overall (n = 329)	Lymphoma (n = 36)	No-lymphoma (n = 293)	χ2/t/Z value	*p*
Diameter/cm		3.11 ± 1.58	2.79 ± 1.12	-1.568‡	0.118
Shape					
Regular	190(57.7%)	25(69.4%)	165(56.3%)	2.265*	0.132
Irregular	139(42.3%)	11(30.6%)	128(43.7%)		
Orientation					
Parallel	293(89.1%)	35(97.2%)	258(88.1%)	1.904†	0.168
Expansive	36(10.9%)	1(2.8%)	35(11.9%)		
Internal echo					
Even	55(16.7%)	7(19.4%)	48(16.3%)	0.216*	0.642
Uneven	274(83.3%)	29(80.6%)	245(83.7%)		
Posterior echo					
Enhancement	240(72.9%)	32(88.9%)	208(70.9%)	5.205*	0.023
NO enhancement	89(27.1%)	4(11.1%)	85(29.1%)		
Cystic components					
Yes	143(43.4%)	8(22.2%)	135(46.1%)	7.423*	0.006
NO	186(56.6%)	28(77.8%)	158(53.9%)		
Calcifications					
Yes	29(8.9%)	1(2.8%)	28(9.6%)	1.086†	0.297
NO	300(91.1%)	35(97.2%)	265(90.4%)		
CDFI					
0	45(13.6%)	0(0%)	45(15.4%)	-3.443§	<0.001
1	115(34.9%)	8(22.2%)	107(36.5%)		
2	93(28.2%)	15(41.7%)	78(26.6%)		
3	76(23.3%)	13(36.1%)	63(21.5%)		

*χ2 test; ^†^, continuous correction χ2 test; ^‡^, independent *T*-test; ^§^, Mann-Whitney U test.

### CEUS characteristics

**Table 4** summarizes and compares the CEUS characteristics of two groups. Specifically, the two groups exhibited statistically significant differences in enhancement degree, enhancement pattern, early wash-in, and early wash-out, whereas no significant differences were observed in enhancement uniformity, enhancement margin, and enhanced lesion size. Salivary gland lymphoma demonstrated a significantly higher proportion of hyperenhancement than non-lymphoma (91.7% vs. 68.3%, *p* = 0.004). Non-centripetal enhancement was also more frequently observed in salivary gland lymphomas (88.9% vs. 44.1%, *p* < 0.00). Furthermore, rapid wash-in and rapid wash-out patterns were significantly more common in salivary gland lymphomas than in non-lymphoma (97.2% vs. 41.6%, *p* < 0.001; 86.1% vs. 40.3%, *p* < 0.001, respectively).

**Table 4:** The CEUS characteristics of lesions in the salivary gland(n=329).

Characteristic	Overall (n = 329)	Lymphoma (n = 36)	No-lymphoma (n = 293)	χ2/t/Z value	*p*
Enhancement degree					
Hyperenhancement	233(70.8%)	33(91.7%)	200(68.3%)	8.500*	0.004
No hyperenhancement	96(29.2%)	3(8.3%)	93(31.7%)		
Enhancement pattern					
Centripetal	168(51.1%)	4(11.1%)	164(55.9%)	25.821*	<0.001
Non-Centripetal	161(48.9%)	32(88.9%)	129(44.1%)		
Enhancement uniformity					
Uniform	34(10.3%)	7(19.4%)	27(9.2%)	2.601†	0.107
Non-uniform	295(89.7%)	29(80.6%)	266(90.8%)		
Enhancement margin					
Clear	207(62.9%)	23(63.9%)	184(62.8%)	0.016*	0.898
Blurred	122(37.1%)	13(36.1%)	109(37.2%)		
Enhanced lesion size					
Expansion	126(38.3%)	15(41.7%)	111(37.9%)	0.194*	0.659
Non-expansion	203(61.7%)	21(58.3%)	182(62.1%)		
Earlier wash-in					
Yes	157(47.7%)	35(97.2%)	122(41.6%)	39.704*	<0.001
No	172(52.3%)	1(2.8%)	171(58.4%)		
Earlier washout					
Yes	149(45.3%)	31(86.1%)	118(40.3%)	27.187*	<0.001
No	180(54.7%)	5(13.9%)	175(59.7%)		

*, χ2 test; ^†^, continuous correction χ2 test.

### The logistic regression analysis of salivary gland lymphomas and the non-lymphomatous lesions

**Figure 7:**
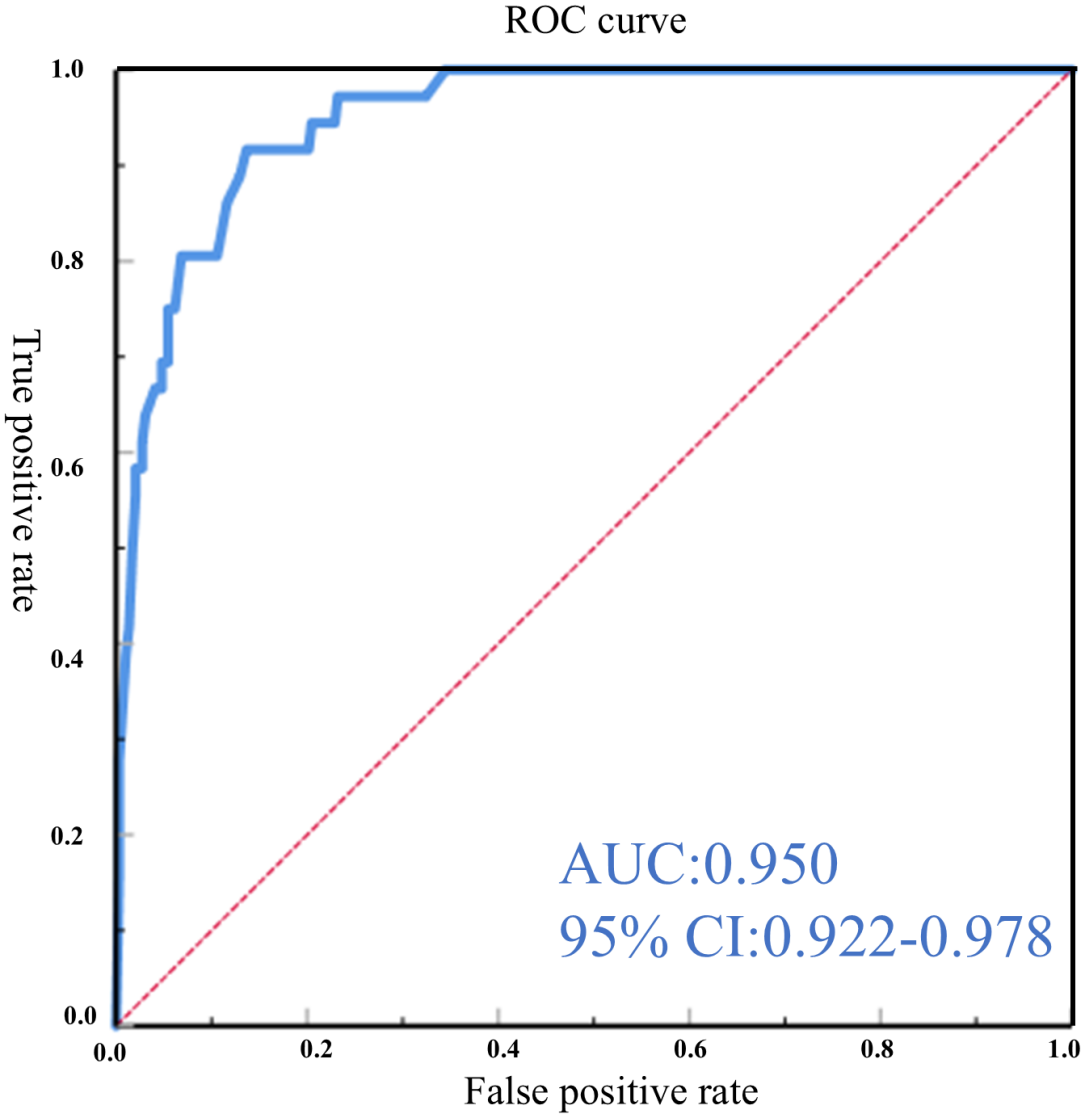
Receiver operating characteristic (ROC) curve of the combined US and CEUS model for differentiating salivary gland lymphoma from non-lymphoma lesions.

**Table 5** presents the results of logistic regression analysis of US and CEUS characteristics associated with salivary gland lesions (n=329). Multivariate analysis identified several independent predictors of salivary gland lymphomas. Lesions presenting with multiple masses (OR = 4.329, 95% CI: 1.382–13.557, *p* = 0.012) or exhibiting posterior echo enhancement (OR = 11.197, 95% CI: 2.975–42.143, *p* < 0.001) showed significantly increased odds of salivary gland lymphomas. Regarding CEUS features, an early wash-in pattern was strongly associated with salivary gland lymphomas (OR = 30.973, 95% CI: 3.655–262.450, p=0.002). Conversely, an early wash-out pattern was also a significant predictor of salivary gland lymphomas (OR = 9.491, 95% CI: 2.614–34.459, *p* = 0.001). Additionally, a non-centripetal enhancement pattern was significantly associated with salivary gland lymphomas (OR = 0.095, 95% CI: 0.027–0.337, *p* < 0.001). The binary logistic regression demonstrated a high specificity of 98.0% (287/293) and an overall accuracy of 93.6% (308/329). The sensitivity for identifying salivary gland lymphoma was 58.3% (21/36). ROC analysis demonstrated that the combined US and CEUS model achieved an area under curve (AUC) of 0.950 (95% CI: 0.922-0.978) (**Figure 7**).

**Table 5:** Logistic regression analysis of lesions in the salivary gland(n=329).

Characteristic	*B*	SE	Wald	*df*	*p*	OR (95%CI)
Number	1.465	0.582	6.330	1	0.012*	4.329(1.382-13.557)
Posterior echo	2.416	0.676	12.759	1	<0.001*	11.197(2.975-42.143)
Cystic components	-1.017	0.580	3.076	1	0.079	0.362(0.116-1.127)
CDFI	0.256	0.309	0.687	1	0.407	1.292(0.705-2.369)
Enhancement degree	0.011	0.769	<0.001	1	0.988	1.001(0.224-4.564)
Enhancement pattern	-2.345	0.645	13.307	1	<0.001*	0.095(0.027-0.337)
Earlier wash-in	3.433	1.090	9.915	1	0.002*	30.973(3.655-262.450)
Earlier washout	2.250	0.658	11.701	1	0.001*	9.491(2.614-34.459)

*p < 0.05.

## 4 Discussion

To the best of our knowledge, few studies have reported data on the preoperative imaging evaluation of salivary gland lymphomas, and there are no reports on the CEUS features of salivary gland lymphoma. In the preoperative evaluation of a newly detected salivary tumor, lymphoma is often not considered in the differential diagnosis (6, 16). US remains the first-line imaging modality for assessing salivary gland lesions, offering the advantages of being non-invasive, easily accessible, and cost-effective, while providing detailed evaluation of superficial anatomical structures. Moreover, US can serve as a guidance tool for biopsy to ensure adequate histological sampling in patients with suspected lymphoma. Notably, salivary gland lymphomas exhibit no specific findings on conventional US. CEUS provides real-time visualization of tumor microvascular perfusion and improves lesion characterization compared with conventional US. Through dynamic visualization of microbubble perfusion, CEUS provides real-time, high-resolution imaging of tumor microvasculature, thereby aiding in the differentiation of benign from malignant lesions (14, 17, 18).

Salivary gland lymphoma is a rare neoplasm, accounting for only 2–5% of all salivary gland tumors (19, 20). Its diagnosis remains challenging, as patients typically present with a painless palpable mass, rendering clinical distinction from benign salivary gland tumors unfeasible (21, 22). To our knowledge, no study has investigated the differential diagnosis between salivary gland lymphoma and non-lymphoma. In this study, we retrospectively analyzed US and CEUS features in 329 salivary gland tumors. Using histopathological examination as the reference standard, several US and CEUS parameters were identified as valuable indicators for differentiating salivary gland lymphoma from non-lymphoma: number, posterior echo, cystic components of masses, CDFI, and CEUS-derived features (enhancement degree, enhancement pattern, early wash-in, and early washout). Binary logistic regression analysis further revealed that lesion multiplicity and posterior acoustic enhancement on US, along with enhancement pattern, early wash-in, and early washout on CEUS, were significant independent predictors for distinguishing salivary gland lymphoma from non-lymphoma. The combined diagnostic model incorporating these predictors yielded a sensitivity of 58.3%, a specificity of 98.0%, and an accuracy of 93.6% in differentiating salivary gland lymphoma from non-lymphoma. Although the combined US and CEUS model showed relatively low sensitivity, its high specificity suggests that CEUS may be more suitable as an adjunctive diagnostic tool rather than a screening modality. In clinical practice, CEUS may improve diagnostic confidence in patients with suspected salivary gland lymphoma and help identify candidates for ultrasound-guided biopsy.

US and CEUS have been widely used to differentiate between benign and malignant salivary gland lesions (23–25). However, studies focusing on distinguishing salivary gland lymphoma from non-lymphoma remain limited. Malignant lesions of salivary gland usually show an irregular shape and rich blood vessels. Our study found no significant differences in characteristics, such as lesion location, shape, internal echogenicity, cystic components, or calcifications. However, salivary gland lymphoma demonstrated a significantly higher prevalence of multiple lesions, posterior echo enhancement, and cystic components compared with non-lymphoma. These findings are consistent with previous reports indicating that over 80% of patients with salivary gland lymphoma present with at least one of the following features: bilateral masses, multiple lesions, or ill-defined margins (22). These data confirm the role of preoperative assessment of salivary gland masses, especially in patients with clinically suspicious findings, and can assist surgeons in deciding whether to perform a parotid gland biopsy rather than a superficial parotidectomy.

There is still a lack of imaging methods that can reliably differentiate salivary gland lesions. Ultrasound is well-suited for locating salivary gland tumors, but it has limited capabilities in differentiating these tumors. Therefore, we used CEUS to describe the distribution of micro-vascularity in the lesion, especially the anechoic areas, and to describe their perfusion dynamics. The CEUS manifestations of lymphoma typically show non-uniform perfusion, hyperenhancement, and an earlier wash-in (26). In our study, hyperenhancement, non-centripetal enhancement, earlier wash-in, and earlier washout were observed significantly more frequently in salivary gland lymphoma than in non-lymphoma, which were statistically significant. Notably, non-centripetal enhancement was present in 80.6% (32/36) of salivary gland lymphoma, compared with only 44.1% (129/293) of non-lymphoma (*p* < 0.05). Recent advances in MRI, with the application of multiparametric protocols, have shown promise in distinguishing salivary gland lymphoma (27, 28). Emerging evidence suggests that ultrasound-guided core needle biopsy (CNB) represents a viable diagnostic alternative for salivary gland lymphoma. Unlike epithelial salivary gland tumors, the primary treatment for salivary gland lymphoma is not surgical resection; therefore, establishing the diagnosis via a minimally invasive approach may obviate the need for surgery (7). Compared with surgical, CNB offers comparable diagnostic accuracy while reducing the risk of complications such as facial nerve injury, sialocele formation, and fistula formation (29). Furthermore, ultrasound guidance enables targeted sampling of suspicious areas, enhancing diagnostic yield in suspected cases of lymphoma.

This study has certain limitations. First, this was a retrospective single-center study, which may have introduced potential selection bias and residual confounding. Second, the sample size of salivary gland lymphoma is substantially fewer than non-lymphoma lesions, reflecting the rarity of salivary gland lymphoma. This class imbalance may have influenced the diagnostic performance of the predictive model, particularly contributing to the relatively high specificity and comparatively low sensitivity. Third, several predictors demonstrated relatively wide confidence intervals, indicating limited statistical stability of the regression estimates. Finally, only qualitative parameters were used in B-mode ultrasound and CEUS, without the application of quantitative indicators.

## 5 Conclusions

In conclusion, US combined with CEUS may improve the differential diagnosis between salivary gland lymphoma and non-lymphoma lesions. Multiple lesions, posterior acoustic enhancement, non-centripetal enhancement, early wash-in, and early wash-out were identified as important imaging indicators of salivary gland lymphoma. CEUS may serve as a valuable adjunctive imaging modality for guiding biopsy decision-making and reducing unnecessary surgical interventions.

## Data Availability

The original contributions presented in the study are included in the article/supplementary material. Further inquiries can be directed to the corresponding author.
